# Prevalence and prognostic value of sarcopenia in patients with bladder cancer undergoing radical cystectomy: a systematic review and meta-analysis

**DOI:** 10.3389/fonc.2025.1642833

**Published:** 2025-09-18

**Authors:** Lei Zhang, Fanmin Li, Lemei Liu, Jianjia Cao, Xiye Yang

**Affiliations:** ^1^ Department of General Medicine, The People’s Hospital of Leshan, Leshan, China; ^2^ Department of Urology, The People’s Hospital of Leshan, Leshan, China

**Keywords:** sarcopenia, bladder cancer, radical cystectomy, prognosis, meta-analysis

## Abstract

**Background:**

The relationship between sarcopenia and clinical outcomes in patients with bladder cancer (BC) has been inconsistently reported in the literature. Some studies have identified sarcopenia as a potential prognostic indicator associated with reduced survival following radical cystectomy (RC).

**Objectives:**

This study was conducted to systematically evaluate the prognostic significance of sarcopenia in patients with bladder cancer undergoing RC.

**Design:**

Systematic review and meta-analysis.

**Methods:**

We conducted a comprehensive search of multiple databases, including PubMed, EMBASE, Web of Science, Cochrane Library, CHINAHL, China National Knowledge Infrastructure (CNKI) Databases, and Wanfang Database, up to August 23, 2023, to identify both retrospective and prospective cohort studies. To assess the methodological quality of the included studies, the Newcastle-Ottawa Scale was utilized to evaluate the risk of bias. Furthermore, heterogeneity and potential publication bias were examined, and both subgroup and sensitivity analyses were performed to ensure the robustness of the findings.

**Results:**

A total of 18 studies comprising 3,110 patients were included in the quantitative synthesis. The results of meta-analysis showed that the pooled prevalence of sarcopenia was estimated to be 49% (95% CI: 41% to 57%, I^2^ = 95.3%, P < 0.001), which was based on a random-effects model. We observed that BC patients with sarcopenia had a worse OS (HR:1.64, 95% CI: 1.30 to 1.97, I^2^ = 76.5%, P < 0.001} and CSS (HR:1.86, 95% CI: 1.45 to 2.27, I^2^ = 0.0%, P < 0.001).

**Conclusion:**

Sarcopenia is commonly observed among patients with bladder cancer and appears to be an important prognostic indicator associated with decreased OS and CSS in those undergoing radical cystectomy. Further prospective studies are warranted to validate these findings.

**Systematic review registration:**

https://www.crd.york.ac.uk/prospero/, identifier CRD42023456724.

## Introduction

Bladder cancer (BC) is the ninth most commonly diagnosed malignancy worldwide, with an estimated annual incidence of approximately 430,000 new cases (1). BC is more prevalent in men and is the fourth most commonly diagnosed cancer among men in industrialized countries, including the United States and Germany (2, 3). Radical cystectomy (RC) is the gold standard treatment for muscle-invasive bladder cancer (MIBC) and for cases of non-muscle-invasive bladder cancer (NMIBC) unresponsive to intravesical therapy (4). Although RC is performed with curative intent, the 5-year overall survival (OS) rate remains relatively low, ranging from approximately 50% to 60% (3, 5). Established factors influencing survival outcomes following radical cystectomy (RC) encompass patient age, histopathological features, and the presence of comorbid conditions (6, 7). While these factors serve as important indicators of overall health status, they lack sufficient precision to guide preoperative clinical decision-making. Consequently, there is a need for a reliable preoperative prognostic marker that can effectively stratify patients to optimize surgical management.

Sarcopenia, the most commonly evaluated body composition parameter, is defined as a reduction in muscle mass, a key factor contributing to frailty (6). Recent studies found that sarcopenia was a predictor for survival in several malignancies including lung cancer, ovarian cancer, colorectal cancer, gastric cancer, pancreatic cancer, and prostate cancer (8–14). Sarcopenia has been shown to be a significant predictor of shorter overall survival (OS) and cancer-specific survival (CSS) (8, 9). Various methods have been used to assess sarcopenia, including the skeletal muscle index (SMI), psoas muscle index (PMI), and total psoas index (TPI), among others (15). SMI is a widely used metric for assessing sarcopenia, calculated by normalizing the total muscle cross-sectional area measured at the third lumbar vertebral level on computed tomography (CT) scans by the patient’s height squared (16).

Many studies regarding the predictive value of sarcopenia in patients with bladder cancer have been conducted (17–19). However, the results of these studies are inconsistent and even controversial. For example, Almarzouq et al. (17) found that sarcopenia did not serve as an independent prognostic factor in patients diagnosed with bladder cancer patients. Conversely, Erdik et al. (19) found that sarcopenia was independently associated with poor outcomes in patients treated with RC. Thus, we conducted a systematic review and meta-analysis to summarize the current evidence regarding the prognostic role of sarcopenia in bladder cancer patients undergoing RC.

## Material

### Protocol and registration

This review was conducted following the Preferred Reporting Items for Systematic Reviews and Meta-Analyses (PRISMA) statement (20) and is registered in PROSPERO (CRD42023456724). The review followed the registered protocol without any deviations.

### Literature search

A comprehensive search of English literature using the database of PubMed, EMBASE, Web of Science, Cochrane Library, CHINAHL, China National Knowledge Infrastructure(CNKI) Databases, Wanfang Database. Screen the reference of the included articles to identify any other eligible studies. The following Mesh terms and keywords were include: ‘bladder’, ‘urothelial carcinoma’, ‘muscle-invasive bladder cancer’, ‘non-muscle-invasive bladder cancer’ ‘ sarcopenia’, ‘skeletal muscle index’, ‘muscle strength’, ‘Psoas muscle index’, the detailed search strategy is shown in **Supplementary Table S1**.

We use the Boolean operators “OR” and “AND” between the groups. The publication year of these articles was limited to January 1, 2008 to September 2023, and only full-text original research articles are considered. All search results are downloaded and imported directly into Zotero, version 6.0.

### Eligibility criteria

We enrolled studies according to the following inclusion criteria: (1) study population: patients with any type of bladder cancer; (2)) indicator: sarcopenia (each study definition was applied, because no unique definition exists); (3) evaluated the prognostic value of preoperative sarcopenia; (4) outcomes: overall survival (OS), cancer-specific survival(CSS), the prevalence of sarcopenia, or other available data of survival; (5) study type: prospective or retrospective studies. The exclusion criteria are: (1) the sample size was less than 50; (2) no available data for analysis; (3) studies with incomplete data, such as prevalence of sarcopenia, incomplete baseline characteristics, or other critical survival data.

### Study selection

The study selection process was conducted by two independent reviewers (Zhang and Liu), who initially screened titles and abstracts to identify potentially eligible articles. Subsequently, a separate pair of reviewers (Li and Cao) independently assessed the full texts to determine study inclusion or exclusion. Reasons for exclusion were documented, and any discrepancies were resolved through discussion. If consensus could not be achieved, a third reviewer was consulted to make the final decision.

### Data extraction

**Figure 1:**
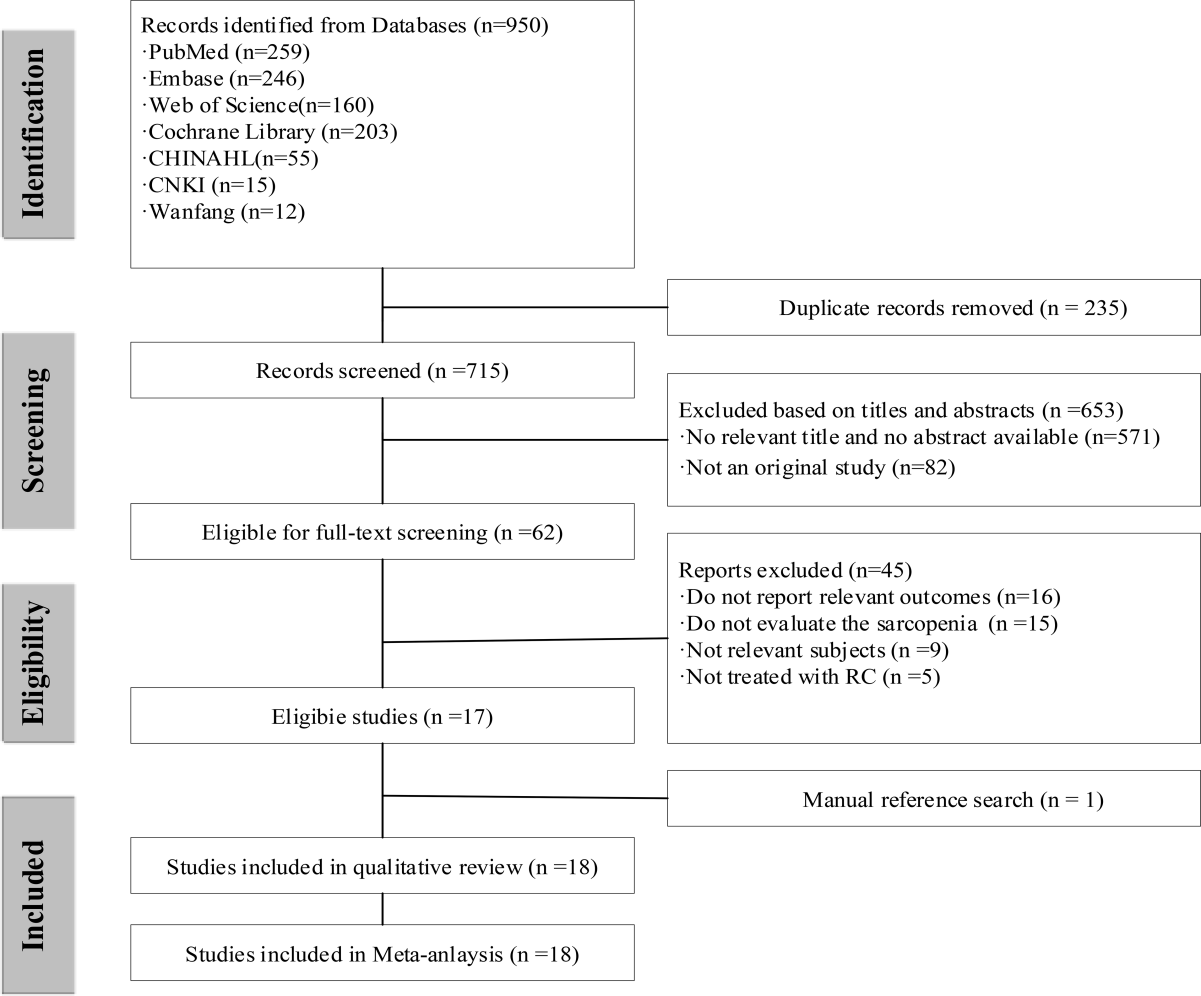
The article selection process.

Two authors (Zhang and Yang) independently extracted relevant data from all eligible studies, including author names, study design, sample size, disease types, treatment modalities, patient age, sarcopenia definitions, and follow-up durations. The extracted datasets were cross-checked, and any discrepancies were resolved through consultation of the original articles. The study selection process is illustrated in the PRISMA flow diagram (**Figure 1**).

### Quality appraisal

Two reviewers independently assessed the risk of bias and overall study quality using the Newcastle-Ottawa Quality Assessment Scale (NOS) (21). The NOS scores range from 0 to 9, with studies categorized as low (0–3), moderate (4–6), or high quality (7–9). Any disagreements were resolved through discussion until consensus was achieved.

### Definition of outcomes

To ensure consistency in the analysis, we have adopted standardized operational definitions for OS and CSS based on widely accepted criteria in the oncology field. OS is defined as the time from the date of diagnosis or treatment initiation to the date of death from any cause, or the last known follow-up date if the patient is still alive. CSS is defined as the time from the date of diagnosis or treatment initiation to the date of death specifically due to cancer, with patients who die from other causes being censored.

Given the variability in definitions across studies, we have taken the following approaches:1. For studies that did not explicitly define OS or CSS or used a non-standard definition, we referred to the most commonly accepted definitions in the literature, as described above. When possible, we consulted with the authors of these studies for clarification to ensure the consistency of the outcomes. 2. For studies where the definition of OS or CSS significantly differed from the standardized definition, we excluded them from the pooled analysis to prevent excessive heterogeneity.

### Data synthesis

For analysis, the results extracted from the included studies were input into Stata 15.0 software package. The endpoints of OS and CSS were characterized by HR and 95%CI, and the pooled prevalence were estimated by proportion. The degree of heterogeneity was tested by Cochrane’s Q test (p value) and the I^2^ statistic (22). We set the I^2^ values as 25%, 50%, and 75%, indicating low, medium, and high heterogeneity, respectively. When no significant heterogeneity was detected (I^2^ ≤ 50), the fixed-effects model was used for pooled prevalence and risk estimates, otherwise the random-effects model was used (23). We also employed funnel plot asymmetry to detect the potential publication bias. An Egger’s regression was applied to test the funnel plot symmetry (24).

Finally, the sensitivity analyses was performed to examine the influence of each study on the pooled estimates of the primary outcome. The data of included studies were divided into subgroups according to regions, measurement and median follow-up time. Due to the limited number of studies in certain subgroup analyses, sensitivity analyses were performed only for meta-analyses comprising more than two studies. All statistical tests were two-sided, with a significance threshold set at P < 0.05.

## Results

### Study selection

In total, 950 related citations (PubMed: 259, EMBASE: 246, Web of science:160, Cochrane Library:160, CINAHL: 55, CNKI:15, Wanfang:12) were identified and qualified through electronic database search, of which 235 were duplicates. After screening titles and abstracts and removing duplicate references, 715 articles were selected on the basis of inclusion criteria. Of these studies, 45 were excluded because of irrelevant outcome and population, not evaluate the sarcopenia, not treated with RC. A total of 18 studies (17–19, 23, 25–38) were utilized in this study. The selection process for the study is shown in the PRISMA flow chart (**Figure 1**).

### Characteristics of the included primary studies

A total of 18 studies were included, all of which were retrospective and included 3,110 patients who were treated with RC. Eligible patients were relatively old, with a median age ranging from 44 to 92 years, and most were from the United States and Japan. In terms of the definition of sarcopenia, most of these studies identified sarcopenia by measuring SMI (17–19, 23, 25–28, 30–32, 34–36, 38) and PMI (29, 33, 37) at the level of the L3 using computed tomography (CT) images, only one study diagnosed based on PMV. The definition of sarcopenia is detailed in **Table 1**. The characteristics of the 18 studies are summarized in **Table 2**.

**Table 1:** The definition of sarcopenia.

Body composition measurement	Definition
SMI (International Consensus) (39)	Women: SMI of <39 cm^2^/m^2^; Men: SMI <55 cm^2^/m^2^
SMI (Martin) (40)	Women: SMI <41 cm^2^/m^2^; Men: SMI<43cm^2^/m^2^ + BMI <25 kg/m^2^; SMI <53 cm^2^/m^2^ and BMI≥25 kg/m^2^
SMI (Yamashita) (36)	Men: SMI <40.8 cm^2^/m^2^; Women: SMI <34.9 cm^2^/m^2^
PMI (Hamaguchi) (41)	Men: PMIs <6.36 cm^2^/m^2^; Women: PMI<3.92 cm^2^/m^2^
PMI (Derstine) (42)	Men: PMIs <7.4 cm^2^/m^2^; Women: PMI<5.2 cm^2^/m^2^
PMV (Zargar) (37)	Psoas muscle volume(PMV) loss>5%

SMI, Skeletal muscle index; PMI, Psoas muscle index; PMV, Psoas muscle volume.

**Table 2:** Characteristics of included studies.

Study	Year	Country	Study design	Simple size	Period of sampling	Age median (IQR)	Sarcopenia definition	Prevalence sarcopenia(%)	Follow-up median IQR (months)	Outcome	NOS
Almarzouq et al. (17)	2021	Canada	Retrospective	141	2002 and 2008	74 (65-81)	SMI (International Consensus)	56.7%	32 months (IQR: 18−66)	OS	7
Borrelli et al. (25)	2023	Italy	Retrospective	97	2018 and 2021	73 (64-74)	SMI (International Consensus)	52.6%	17.43 months (IQR: 1.6−80.9)	OS	8
Engelmann1 et al. (18)	2023	Germany	Retrospective	657	Aug 2004 and Dec 2020	70 (63-77)	SMI (International Consensus)	50.0%	40months (IQR: 15−76)	OS,CSS	8
Erdik et al. (19)	2023	Turkey	Retrospective	84	Sep 2012 and Jun 2020	69 (48-92)	SMI(Martin)	53.6%	70 months (IQR: 60−111)	OS,CSS	7
Fraisse et al. (26)	2019	France	Retrospective	146	Jun 2012 and Apr 2017	66 (44-84)	SMI(Martin)	42.8%	20.4 months (IQR:16-33)	OS	7
Ha et al. (27)	2021	Korea	Retrospective	80	Aug 2008 and May 2013	66 (64-74)	SMI(Martin)	47.5%	46 months	OS	8
Hirasawa et al. (28)	2016	Japan	Retrospective	136	Mar 2003 and Jan 2015	71 (10.3)	SMI(Martin)	76%	32 months (IQR: 18−66)	CSS	7
Lyon et al. (23)	2019	United States	Retrospective	177	2000 and 2016	65 (57-72)	SMI (International Consensus)	55.0%	36 months (IQR: 20−60)	CSS	8
Miyake et al. (29)	2017	Japan	Retrospective	117	Jan 2006 and Jul 2016	72 (61-77)	PMI(Hamaguchi)	20.0%	22 months (IQR: 10−24)	OS	7
Miyake et al. (30)	2016	Japan	Retrospective	89	Jan 2006 and Oct 2014	71 (48-83)	SMI(Martin)	25.0%	29 months (IQR: 10−60)	OS,CSS	7
Psutka et al. (32)	2014	United States	Retrospective	205	2000 and 2007	71 (63-78)	SMI (International Consensus)	55.6%	76 months (IQR:71-122)	OS,CSS	7
Psutka et al. (31)	2015	United States	Retrospective	262	2000 and 2008	71 (65-81)	SMI (International Consensus)	67.6%	75months (68-114)	OS	6
Kremser et al. (33)	2021	United States	Retrospective	441	2007 and 2012	68 (59-75)	PMI(Derstine)	32.7%	14 months (IQR: 6−23)	OS	7
Taguchi et al. (34)	2015	Japan	Retrospective	64	Apr 2003 and Feb 2014	68 (63-73)	SMI (International Consensus)	–	13 months (IQR: 9−26)	CSS	8
Wang et al. (35)	2021	China	Retrospective	112	Jan 2012 and Dec 2020	65 (10.6)	SMI (International Consensus)	59.8%	22 months (IQR: 10−24)	OS	7
Yamashita et al. (36)	2020	Japan	Retrospective	123	Jul 2010 and Feb 2019	74 (69-79)	SMI(Yamashita)	39%	39 months (IQR: 21−63)	OS,CSS	7
Zargar et al. (37)	2017	United States	Retrospective	130	Jan 2009 and Dec 2013	62 (54-70)	PMV(Zargar)	55.6%	15.5 months (IQR:8-23)	OS,CSS	6
Mao et al. (38)	2020	China	Retrospective	200	Mar 2009 and Oct 2018	66 (10.1)	SMI (International Consensus)	33.5%	75months (68-114)	OS	6

**Quality assessment**

The quality assessment and risk of bias were conducted in accordance with the NOS. The NOS score of each included study is listed in **Table 1**; All studies evaluated were of high quality (NOS score ≥ 6).The detailed quality assessment results are displayed in **Supplementary Table S2**.

**Prevalence of sarcopenia**

**Figure 2:**
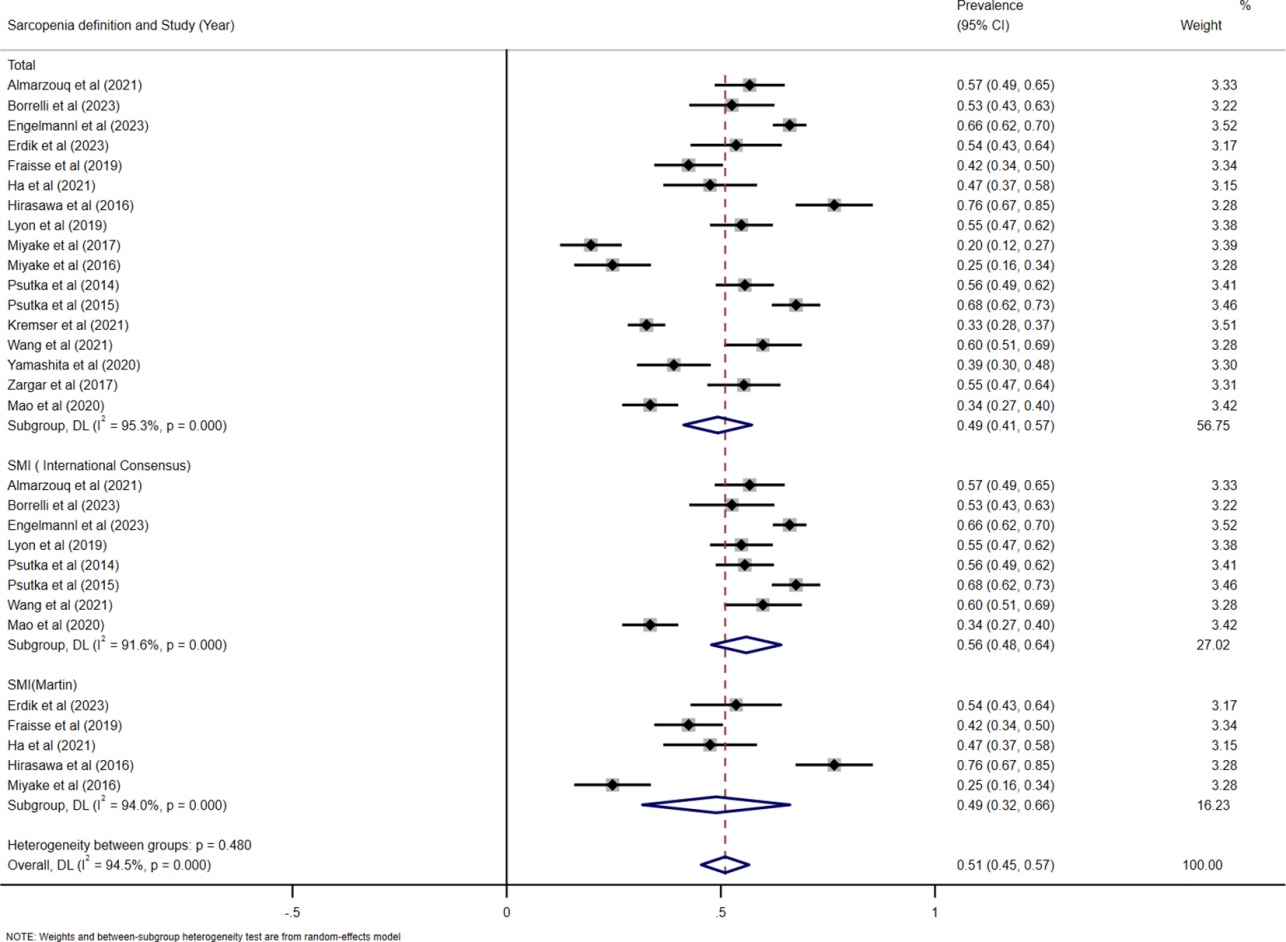
Forest plots for the pooled prevalence of sarcopenia in patients with MIBC.

**Figure 3:**
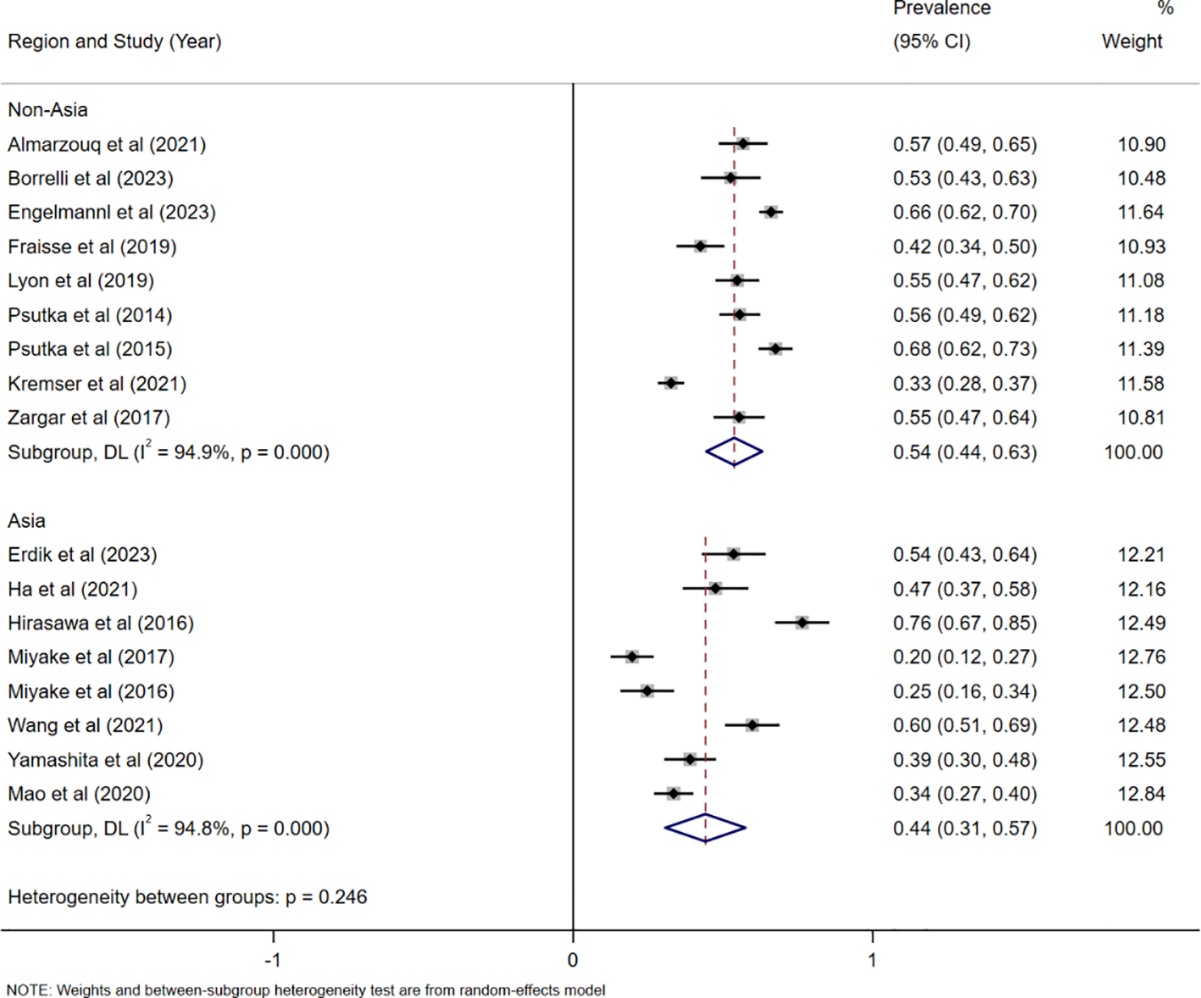
Forest plots for the pooled prevalence of sarcopenia stratified by country.

In the 18 studies available for the meta-analysis, the pooled prevalence of sarcopenia was estimated to be 49% (95% CI: 41% to 57%, I^2^ = 95.3%, P < 0.001), which was based on a random-effects model. Among them, the prevalence of sarcopenia defined by SMI (International Consensus) was estimated to be 56% (95% CI: 48% to 64%, I^2^ = 91.6%, P < 0.001), the sarcopenia defined as SMI(Martin) was estimated to be 49% (95% CI: 32% to 66%, I^2^ = 94%, P < 0.001)(**Figure 2**). In addition, a stratified analysis was conducted according to regions, and the results are shown in **Figure 3**.

### Effects of sarcopenia on overall survival

**Figure 4:**
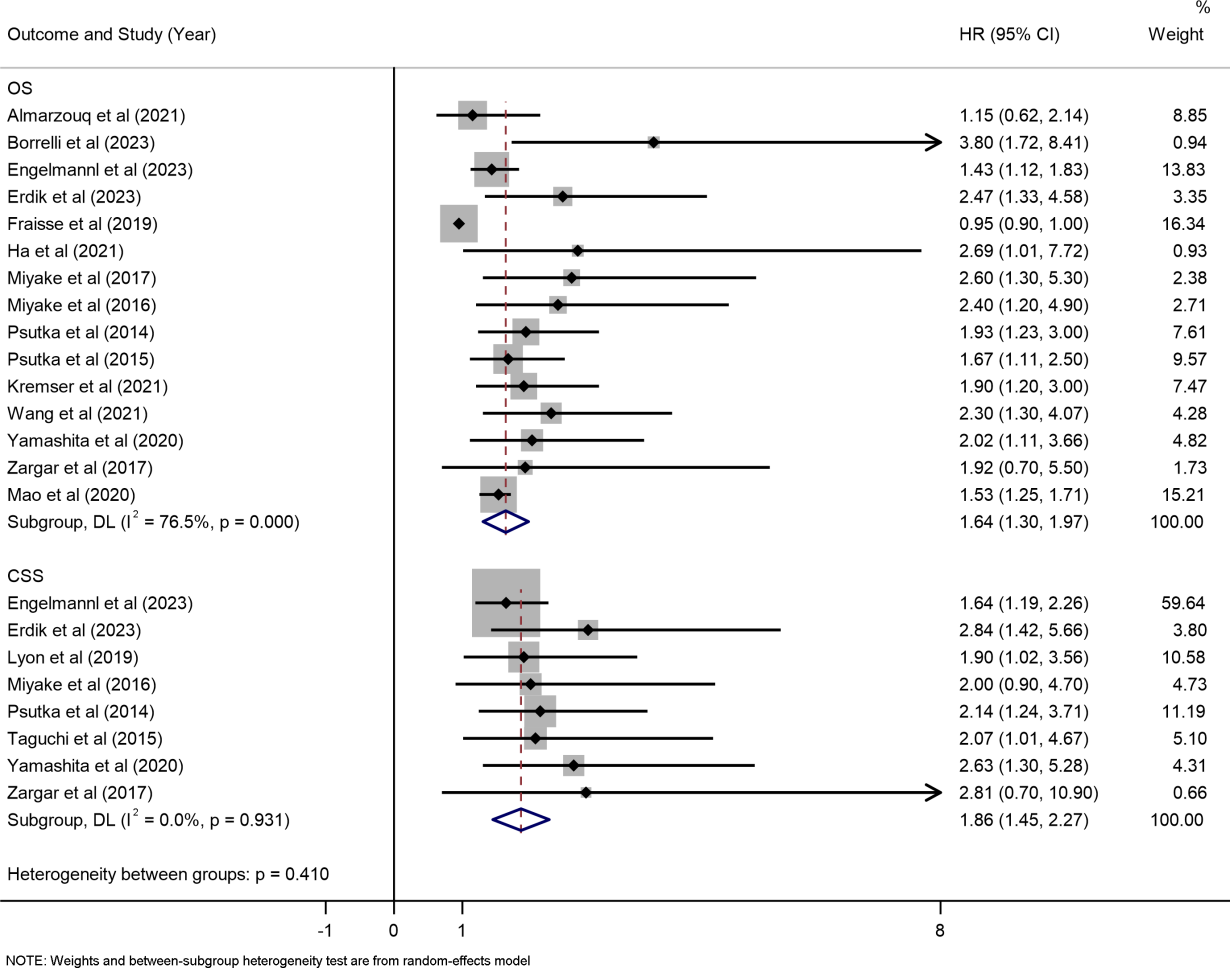
Forest plot of the hazard ratios of sarcopenia for overall survival and cancer-specific survival.

Data from 15 studies, including 2,784 participants, were available to meta-analyze overall survival. Of the included studies, OS was defined in 12 from treatment initiation to death, or the last follow-up (15, 17–19, 26, 27, 29–33, 37); and from the time of diagnosis to death or the last follow-up, in the other 3 studies (25, 35, 38), OS was not clearly defined in the remaining two studies. We observed that patients with sarcopenia had a worse OS compared with those without sarcopenia, the pooled HR was 1.64 (95% CI: 1.30 to 1.97, I^2^ = 76.5%, P < 0.001; **Figure 4**). Because of high heterogeneity was revealed, so we used the random-effect model.

### Effects of sarcopenia on cancer-specific survival

Data from 9 studies, including 1514 participants, were available to meta-analyze cancer-specific survival. Of the included studies, CSS in 7 studies was defined as the interval from RC to death attributable to bladder cancer progression or metastasis. CSS was not clearly defined in the remaining two studies. The results of meta-analysis showed that sarcopenia was associated with poor CSS, the pooled HR is 1.86 (95% CI: 1.45 to 2.27, I^2^ = 0.0%, P < 0.001; **Figure 4**).

### Subgroup analyses

We conducted subgroup analyses based on geographical region, measurement methods, and follow-up time separately for the outcomes of OS and CSS, as shown in **Table 3**. The studies were divided into two groups: Asian and non-Asian regions, with 7 and 8 studies included, respectively. The meta-analysis results revealed that sarcopenia was a predictive factor associated with a decrease in both OS (HR: 1.55, 95% CI: 1.45 to 2.27, P < 0.001; HR: 1.45, 95% CI: 1.06 to 1.84, P < 0.002) and CSS (HR: 2.34, 95% CI: 1.50 to 3.17, P < 0.001; HR: 1.75, 95% CI: 1.30 to 2.21, P < 0.001), irrespective of whether the cases originated from Asian or non-Asian populations.

**Table 3:** Subgroup analyses of OS and CSS.

Subgroups	Variable	Number of include studies	HR (95%CI)	Sarcopenia		
				Pooling model	*P* value	*I²(%)*
OS						
Regions	Asia	7	1.55 (1.36-1.74)	Fixed	<0.001	0%
	Non-Asia	8	1.45 (1.06-1.84)	Random	<0.002	69.8%
Measurement	SMI (International Consensus)	7	1.53 (1.35-1.70)	Fixed	<0.001	0%
	SMI(Martin)	4	1.75 (0.66-2.83)	Random	0.080	55.5%
Follow-up	≥ol month	8	1.53 (1.36,1.71)	Fixed	<0.001	0%
	<30 month	7	1.89 (1.12-2.67)	Random	<0.013	63.0%
CSS						
Regions	Asia	5	2.34 (1.50-3.17)	Fixed	<0.001	0%
	Non-Asia	4	1.75 (1.30-2.21)	Random	<0.001	0%
Measurement	SMI (International Consensus)	4	1.76 (1.32-2.21)	Fixed	<0.001	0%
	SMI(Martin)	3	2.34 (1.28-3.4)	Fixed	<0.001	0%
Follow-up	≥ol month	5	1.88 (1.44,2.29)	Fixed	<0.001	0%
	<30 month	3	2.08 (0.81-3.36)	Fixed	0.187	0%

In the subgroup analysis based on measurement methods, due to limited study numbers, we only conducted meta-analyses for SMI (International Consensus) and SMI(Martin). The results showed that sarcopenia measured using SMI (International Consensus) was correlated with a decrease in OS (HR: 1.53, 95% CI: 1.35 to 1.70, P < 0.001), while SMI(Martin) did not reach statistical significance (HR: 1.75, 95% CI: 0.66 to 2.83, P < 0.080). Both measurement methods, SMI (International Consensus) and SMI(Martin), had an impact on the decrease in CSS (HR: 1.76, 95% CI: 1.32 to 2.21, P < 0.001; HR: 2.34, 95% CI: 1.28 to 3.40, P < 0.001).

Regarding median follow-up time, we used a cut-off of 30 months for subgroup analysis. The results showed that sarcopenia was associated with a decrease in OS regardless of whether the median follow-up time exceeded 30 months (HR: 1.53, 95% CI: 1.36 to 1.71, P < 0.001; HR: 1.89, 95% CI: 1.12 to 2.67, P < 0.013). For CSS, subgroup analysis with a median follow-up time exceeding 30 months revealed an association with sarcopenia (HR: 1.88, 95% CI: 1.44 to 2.29), but no statistically significant association was observed in the subgroup with a median follow-up time of less than 30 months (HR: 2.08, 95% CI: 0.81 to 3.36, P < 0.187). Forest plots for all outcomes are provided in **Supplementary Figures 5**-**10**.

### Sensitivity analyses and publication bias

To evaluate the robustness and reliability of the primary analysis, sensitivity analyses were conducted by sequentially excluding individual studies. The results indicated that the overall survival outcomes remained consistent regardless of the removal of any single study, including those of relatively lower methodological quality. The results are shown in **Supplementary Figure 1**.

Publication bias was assessed using Begg’s test, and funnel plots were examined for symmetry. The P-values for Begg’s test were 0.223 for OS and 0.978 for CSS, suggesting no significant evidence of publication bias in the meta-analysis. The funnel plots are provided in the **Supplementary Materials**.

## Discussion

This meta-analysis of 18 studies including 3,110 patients aimed to determine the predictive value of sarcopenia for prognosis in patients treated with RC. Our results showed that the pooled prevalence of sarcopenia defined as SMI (International Consensus) was 56% (95% CI: 48% to 64%, I^2^ = 91.6%), and the pooled prevalence of sarcopenia defined as SMI(Martin) was 49% (95% CI: 32% to 66%, I^2^ = 94%). The high heterogeneity observed in both prevalence estimates could be attributed to several factors, including variations in patient populations (e.g., age, comorbidities, cancer stage) and regional differences in diagnostic practices. In addition, we observed a higher prevalence of sarcopenia in samples from Asia than from non-Asia(44% VS.54%), but this result may be confounded by the different measurement due to the limited number of studies. Other factors such as comorbidities (e.g., diabetes, cardiovascular diseases), treatment variations (e.g., neoadjuvant chemotherapy, radiation therapy), and the patient’s functional status could potentially confound the observed relationship between sarcopenia and survival outcomes. These factors may alter the survival prognosis in bladder cancer patients undergoing RC, and future studies should consider controlling for these potential confounders to better clarify the independent effect of sarcopenia on OS and CSS.

In accordance with the results of the meta-analysis, the forest plots clearly demonstrated that sarcopenia could significantly predict worse OS and CSS. The association between sarcopenia and decreased survival has been previously described in various malignancies. For instance, Peng et al. (43) reported an independent correlation between sarcopenia and an increased risk of all-cause mortality in a cohort of 296 patients who had undergone surgical resection for pancreatic cancer. Notably, patients without sarcopenia had a median overall survival of 18 months, compared to 13.7 months in those with sarcopenia (P < 0.01). Furthermore, multivariable analysis revealed that sarcopenia was associated with a 67% increased risk of all-cause mortality at 3 years ([HR]: 1.67; 95% CI: 1.28–2.07; P < 0.001).Similarly, Harimoto et al. (44) reported a significantly lower OS rate in sarcopenic patients undergoing partial hepatectomy for hepatocellular carcinoma compared to non-sarcopenic patients (71% vs. 83.7%; P = 0.001). Furthermore, in the series by Martin et al. (40) series involving 1471 patients afflicted with gastrointestinal or respiratory tract malignancies, patients with sarcopenia had a median OS of 13.0 months, contrasting with the 20.1 months observed in those with normal SMI. Comparable adverse impacts of sarcopenia on OS have also been documented in patients with pancreatic, lung, and colorectal cancers. To our knowledge, few studies have specifically addressed the impact of sarcopenia on CSS. Nonetheless, inferior oncological outcomes have been observed among patients with hepatobiliary cancer24 and melanoma,16 where sarcopenia was noted as a contributing factor.

Moreover, we methodically stratified the dataset based on geographic region, the measurement of sarcopenia, and the median duration of follow-up. The subgroup analyses consistently underscored the significant statistical association between sarcopenia and OS following RC. Given the disparities inherent to the Asian and Western populations, we conducted an evaluation of the relationship between sarcopenia and OS in two regions. The results demonstrate that sarcopenia was independently associated with increased risks of postoperative CSS in both the Asian and non-Asian subsets, which was consistent with previous research endeavors. For example, Miyake et al. (30) examined postoperative cystectomy for bladder carcinoma in Japan. Their work substantiates the assertion that sarcopenia status at baseline and a ≤-10% loss in the psoas muscle were identified as independent prognostic factors for overall survival. A study conducted in Korea by Ha et al. (27) similarly reported that the overall mortality rate was significantly higher in patients with sarcopenia than in those without sarcopenia 1 year after RC. Taking into consideration that the duration of follow-up can potentially introduce bias into the study outcomes, we categorized all studies into two groups using a threshold of 30 months for analysis. The results consistently indicate that sarcopenia remains a significant risk factor for OS, regardless of whether the median follow-up time exceeds 30 months. It is noteworthy that when we used Martin’s criteria (40) as the diagnostic standard for sarcopenia, no statistically significant association between sarcopenia and OS was observed. In contrast to the international consensus, Martin’s definition of sarcopenia incorporates not only the SMI but also factors such as gender and BMI when considering a patient’s condition. However, the generalizability of his conclusions remains contentious, primarily due to the retrospective nature of the study. Furthermore, the study cohort consists of Canadian gastrointestinal and lung cancer patients, potentially leading to thresholds that differ from those applicable to bladder cancer patients or individuals with other medical conditions. This discrepancy may impede its feasibility for simplified utilization in routine clinical practice. The findings from Fraisse et al.’s study similarly reported that sarcopenia was not significantly associated with OS and complications.

Our analysis identified a significant association between sarcopenia and reduced CSS, regardless of whether sarcopenia was defined according to the international consensus criteria or Martin’s definition. Likewise, we performed a subgroup analysis of the predictive value of Sarcopenia for CSS in patients with bladder cancer. No disparities were observed between populations in Asian and non-Asian regions, as sarcopenia exhibited significant predictive value for CSS in both groups. Nevertheless, within the subgroups categorized by follow-up duration, we did not identify statistically significant findings for the groups with a median follow-up time of less than 30 months. This may be attributed to the limited inclusion of studies in this group, and further confirmation of these results necessitates additional cohort studies in the future. Yamashita et al. (36) investigated the prognostic relevance of preoperative muscle depletion—including both sarcopenia and myosteatosis—in patients undergoing RC for bladder cancer. Their findings indicated that sarcopenia was an independent and significant predictor of reduced CSS. Similarly, Psutka et al. (32) performed a retrospective cohort study involving 205 patients who underwent RC. Baseline characteristics, including sex, Charlson Comorbidity Index, American Society of Anesthesiologists score, Eastern Cooperative Oncology Group performance status, receipt of neoadjuvant chemotherapy, TNM stage, and tumor grade, were comparable between sarcopenic and non-sarcopenic patients (P > 0.05 for all). However, sarcopenic patients demonstrated significantly worse 5-year CSS compared to their non-sarcopenic counterparts (49% vs. 72%; P = 0.003). Furthermore, sarcopenia was independently associated with an increased risk of cancer-specific mortality (HR: 2.14; P = 0.007).

In summary, our systematic review reinforces the clinical significance of sarcopenia in patients undergoing RC for bladder cancer, as it serves as a noteworthy predictive factor for both overall survival OS and CSS. The identification of sarcopenia as a predictor for OS and CSS carries important clinical implications. Early screening for sarcopenia in bladder cancer patients could help clinicians make more informed treatment decisions, such as incorporating preoperative nutritional optimization, physical therapy, or sarcopenia-related interventions. This could improve postoperative recovery and long-term survival outcomes for these patients. Nevertheless, an undeniable issue persists in the field—there remains a lack of consensus regarding the definition of sarcopenia. Despite all included studies diagnosing sarcopenia through CT scans, variations in measurement criteria across multiple studies continue to pose a substantial hindrance to inter-study comparisons. Further diagnostic research is imperative for determining the optimal criteria for CT scan-based diagnosis of sarcopenia in bladder cancer patients. Furthermore, in future research within this field, it is essential to not only report patients’ survival outcomes but also specify the start and end times of follow-up periods for both OS and CSS. Additionally, focus should be directed toward aspects such as postoperative care management, surgical complications, cancer-related fatigue, and quality of life, among other pertinent factors. This approach will reduce heterogeneity between studies and better assess the prognostic value of sarcopenia in bladder cancer.

### Limitations

While this meta-analysis consolidates current evidence and highlights sarcopenia as an important prognostic indicator in BC, several limitations should be acknowledged. First, substantial clinical heterogeneity among the included studies limited the feasibility of performing meta-analyses for several outcome measures. Second, the diverse definitions of sarcopenia employed across the studies, despite primarily relying on CT scans, exhibited variations in scanning levels and thresholds. Such differences between studies may have biased our results. Likewise, inconsistencies in the definitions of OS and CSS may introduce bias in the pooled results. Third, all included studies were retrospective in nature, which may have introduced a higher risk of bias and contributed to the observed inter-study heterogeneity. Furthermore, it is important to note that all of the included studies were published in English or Chinese, which may have led to language bias. Consequently, the findings should be interpreted with caution, and their applicability to clinical practice remains limited. Prospective, well-designed studies are warranted to further validate the prognostic significance of sarcopenia.

## Conclusion

Sarcopenia is highly prevalent in patients with bladder cancer and emerges as a significant prognostic factor for impaired OS and CSS in BC patients undergoing RC. Further diagnostic research is imperative for determining the optimal criteria for CT scan-based diagnosis of sarcopenia in bladder cancer patients. More prospective studies are required to confirm our findings.

## Data Availability

The original contributions presented in the study are included in the article/**Supplementary Material**. Further inquiries can be directed to the corresponding author.
