# Supplementary material for: Prevalence and prognostic value of sarcopenia in patients with bladder cancer undergoing radical cystectomy: a systematic review and meta-analysis
Source: Front Oncol. 2025 Sep 18;15:1642833. doi: 10.3389/fonc.2025.1642833 (PMC12488412; doi:10.3389/fonc.2025.1642833)
Supplement: Supplementary file 1 [file Table1.docx]

| **Table S1. Characteristics of included studies** | |
| --- | --- |
| Database | Search Strategy |
| PubMed | ① ("bladder" OR "urothelial carcinoma" OR "muscle-invasive bladder cancer" OR "non-muscle-invasive bladder cancer") |
|  | ② ("sarcopenia" OR "skeletal muscle index" OR "muscle strength" OR "Psoas muscle index") |
|  | ③ ("prevalence" OR "prognostic value" OR "risk factors" OR "predictive factors" OR "associated factors") |
|  | ④ #1 AND #2 AND #3 |
| Web of Science | ① ("bladder" OR "urothelial carcinoma" OR "muscle-invasive bladder cancer" OR "non-muscle-invasive bladder cancer") |
|  | ② ("sarcopenia" OR "skeletal muscle index" OR "muscle strength" OR "Psoas muscle index") |
|  | ③ ("prevalence" OR "prognostic value" OR "risk factors" OR "predictive factors" OR "associated factors") |
|  | ④ #1 AND #2 AND #3 |
| Embase | ① ('bladder'/exp OR 'urothelial carcinoma'/exp OR 'muscle-invasive bladder cancer'/exp OR 'non-muscle-invasive bladder cancer'/exp) |
|  | ② ('sarcopenia'/exp OR 'skeletal muscle index'/exp OR 'muscle strength'/exp OR 'Psoas muscle index'/exp) |
|  | ③ ('prevalence'/exp OR 'prognostic value'/exp OR 'risk factors'/exp OR 'predictive factors'/exp OR 'associated factors'/exp) |
|  | ④ #1 AND #2 AND #3 |
| Cochrane Library | ① (MeSH descriptor: [Bladder Neoplasms] explode all trees) |
|  | ② (MeSH descriptor: [Sarcopenia] explode all trees) |
|  | ③ (bladder OR urothelial carcinoma OR muscle-invasive bladder cancer OR non-muscle-invasive bladder cancer) |
|  | ④ (skeletal muscle index OR muscle strength OR Psoas muscle index) |
|  | ⑤ #1 AND #2 AND #3 AND #4 |
| CNKI | ① (膀胱癌 OR 尿路上皮癌 OR 肌肉浸润性膀胱癌 OR 非肌肉浸润性膀胱癌) |
|  | ② (肌肉减少症 OR 骨骼肌指数 OR 肌肉力量 OR 腰大肌指数) |
|  | ③ (流行率 OR 预后价值 OR 危险因素 OR 预测因素 OR 相关因素) |
|  | ④ #1 AND #2 AND #3 |
| Wanfang Database | ① (膀胱癌 OR 尿路上皮癌 OR 肌肉浸润性膀胱癌 OR 非肌肉浸润性膀胱癌) |
|  | ② (肌肉减少症 OR 骨骼肌指数 OR 肌肉力量 OR 腰大肌指数) |
|  | ③ (流行率 OR 预后价值 OR 危险因素 OR 预测因素 OR 相关因素) |
|  | ④ #1 AND #2 AND #3 |
| VIP Database | ① (膀胱癌 OR 尿路上皮癌 OR 肌肉浸润性膀胱癌 OR 非肌肉浸润性膀胱癌) |
|  | ② (肌肉减少症 OR 骨骼肌指数 OR 肌肉力量 OR 腰大肌指数) |
|  | ③ (流行率 OR 预后价值 OR 危险因素 OR 预测因素 OR 相关因素) |
|  | ④ #1 AND #2 AND #3 |

| **Table S2**: Result of the Newcastle-Ottawa scale quality assessment. | | | | | | | | | |
| --- | --- | --- | --- | --- | --- | --- | --- | --- | --- |
| Newcastle-Ottawa scale | Selection (1) |  |  |  | Comparability (2) | Outcome (3) |  |  | Total |
|  | Representativeness  of the exposed cohort | Selection of the non-exposed cohort | Ascertainment of exposure | Outcomes were not present at study initiation | Comparability of cohorts on the basis of the design or analysis | Assessment of outcome | Was follow-up long enough for outcome to occur | Adequacy  of follow-up |  |
| Almarzouq et al.2021 | 1 | 1 | 1 | 1 | 1 | 1 | 0 | 1 | 7 |
| Borrelli et al. 2023 | 1 | 1 | 1 | 1 | 1 | 2 | 0 | 1 | 8 |
| Engelmann1 et al. 2023 | 2 | 1 | 1 | 0 | 1 | 2 | 0 | 1 | 8 |
| Erdik et al. 2023 | 1 | 1 | 1 | 0 | 1 | 2 | 0 | 1 | 7 |
| Fraisse et al. 2019 | 1 | 1 | 0 | 1 | 1 | 2 | 0 | 1 | 7 |
| Ha et al. 2021 | 0 | 0 | 0 | 1 | 2 | 2 | 0 | 1 | 8 |
| Hirasawa et al. 2016 | 1 | 1 | 1 | 1 | 1 | 2 | 0 | 1 | 7 |
| Lyon et al. 2019 | 1 | 1 | 0 | 1 | 2 | 1 | 1 | 1 | 8 |
| Miyake et al. 2017 | 1 | 1 | 1 | 1 | 1 | 1 | 0 | 1 | 7 |
| Miyake et al. 2016 | 1 | 1 | 1 | 1 | 1 | 2 | 0 | 0 | 7 |
| Psutka et al. 2014 | 1 | 1 | 1 | 1 | 1 | 1 | 1 | 0 | 7 |
| Psutka et al. 2015 | 1 | 1 | 1 | 1 | 1 | 1 | 0 | 0 | 6 |
| Kremser et al. 2021 | 1 | 1 | 1 | 1 | 1 | 2 | 0 | 0 | 7 |
| Taguchi et al. 2015 | 1 | 1 | 1 | 1 | 2 | 2 | 1 | 0 | 8 |
| Wang et al. 2021 | 1 | 1 | 1 | 1 | 2 | 1 | 0 | 0 | 7 |
| Yamashita et al. 2020 | 1 | 1 | 1 | 1 | 1 | 2 | 0 | 0 | 7 |
| Zargar et al. 2017 | 1 | 1 | 1 | 1 | 1 | 1 | 0 | 0 | 6 |
| Mao et al. 2020 | 0 | 1 | 0 | 1 | 1 | 1 | 1 | 1 | 6 |


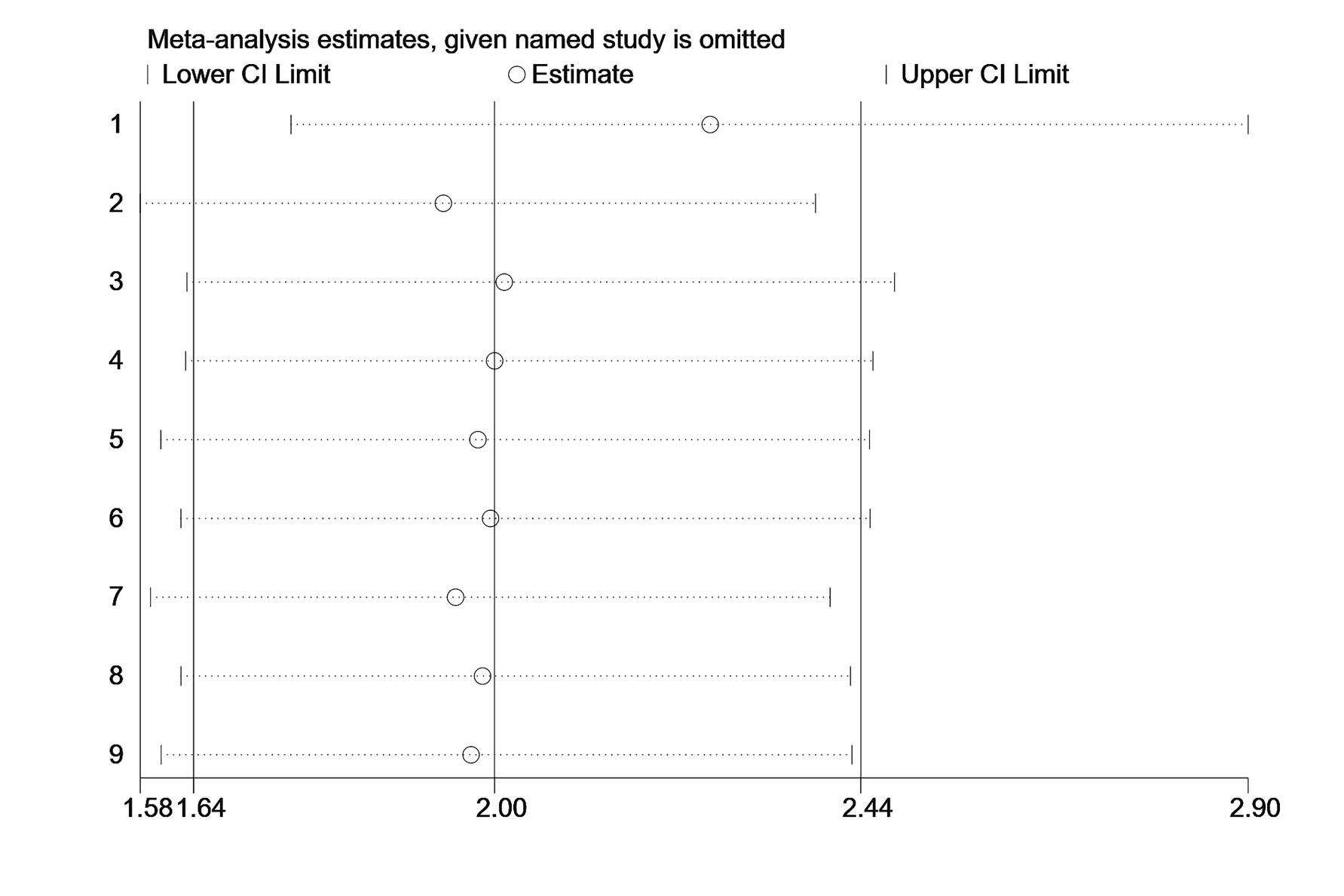


Fig S1.Sensitivity analysis for meta-analysis of sarcopenia for CSS


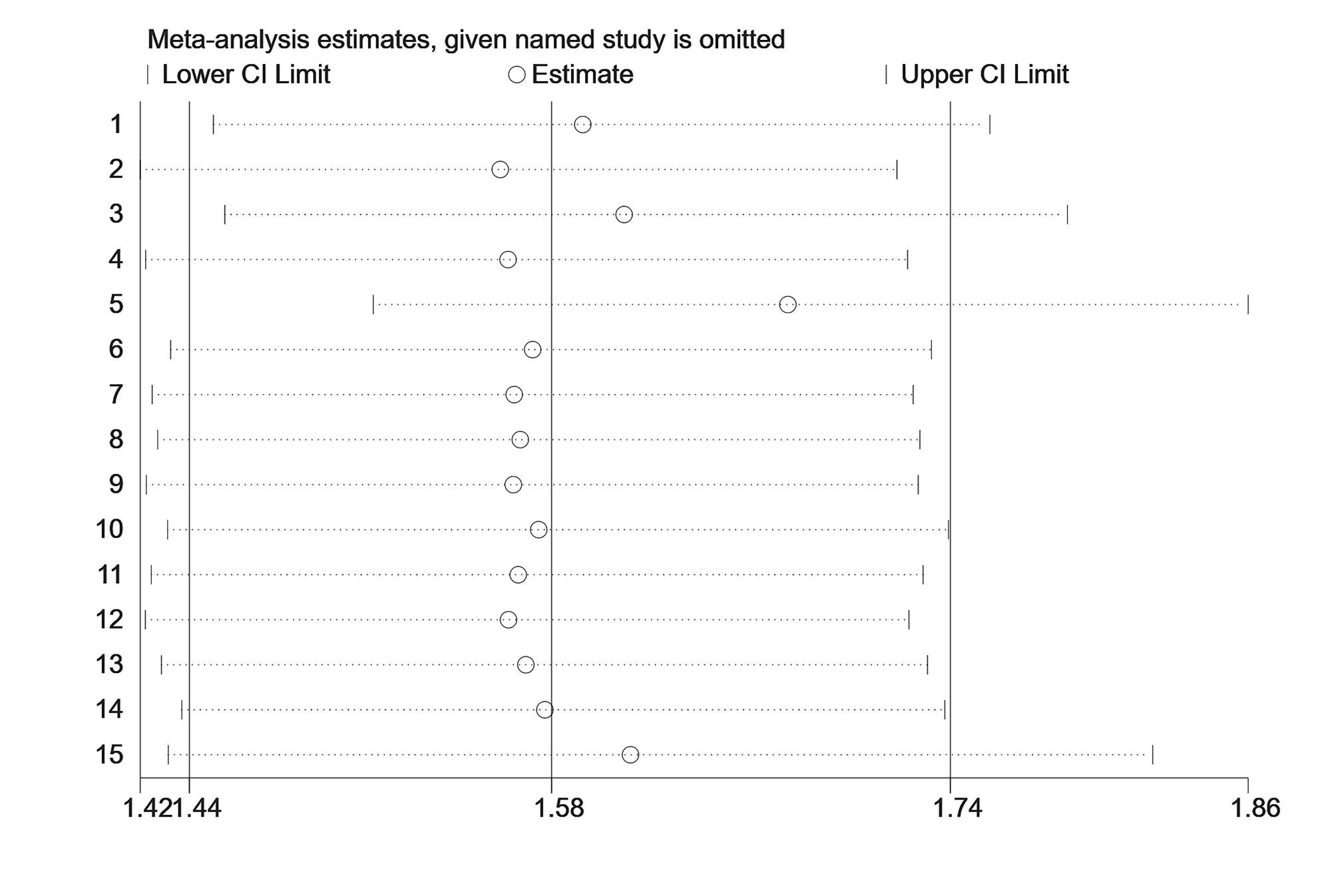
Fig S2.Sensitivity analysis for meta-analysis of sarcopenia for OS


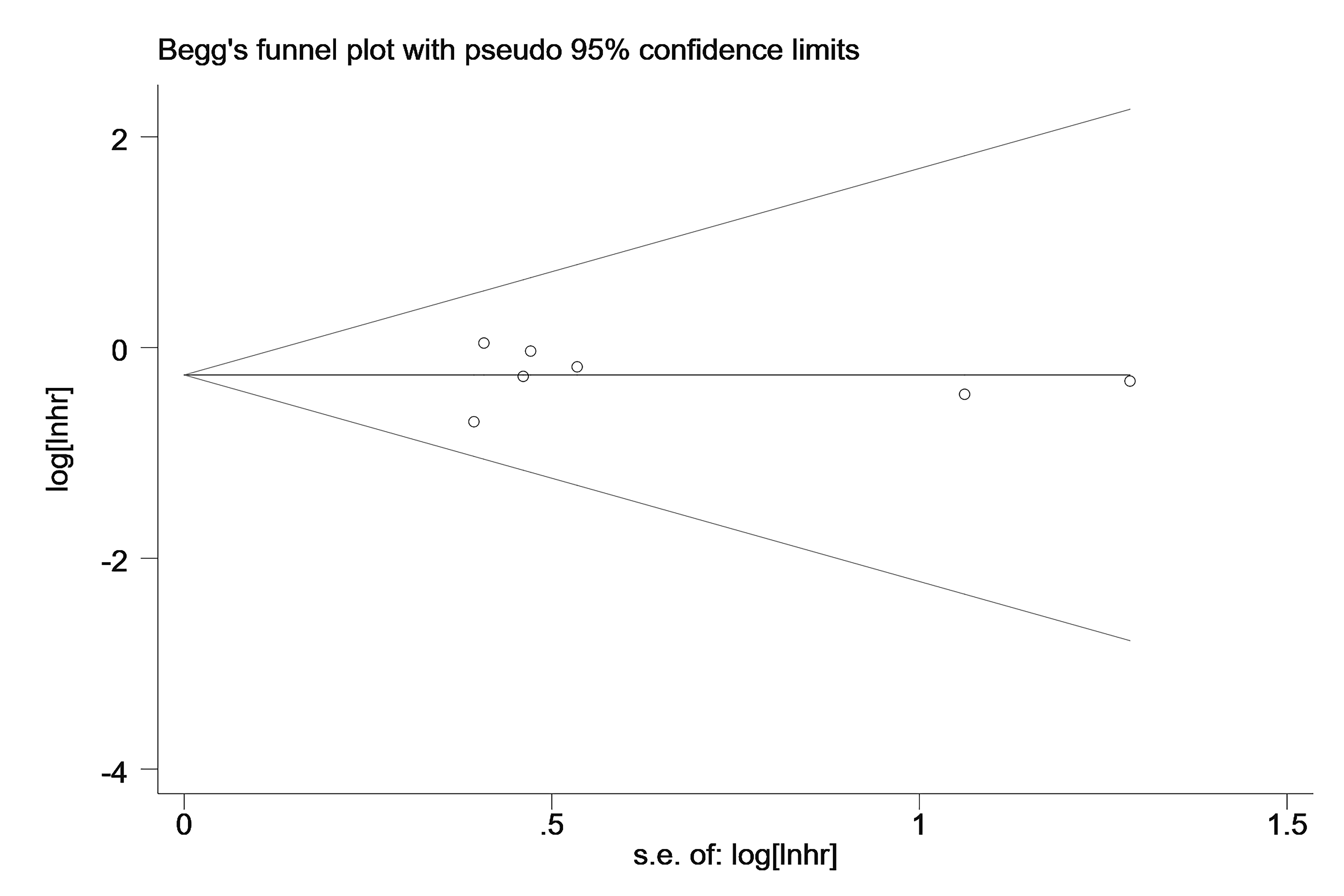


Fig S3. Funnel plots of publication bias for meta-analysis of sarcopenia for CSS


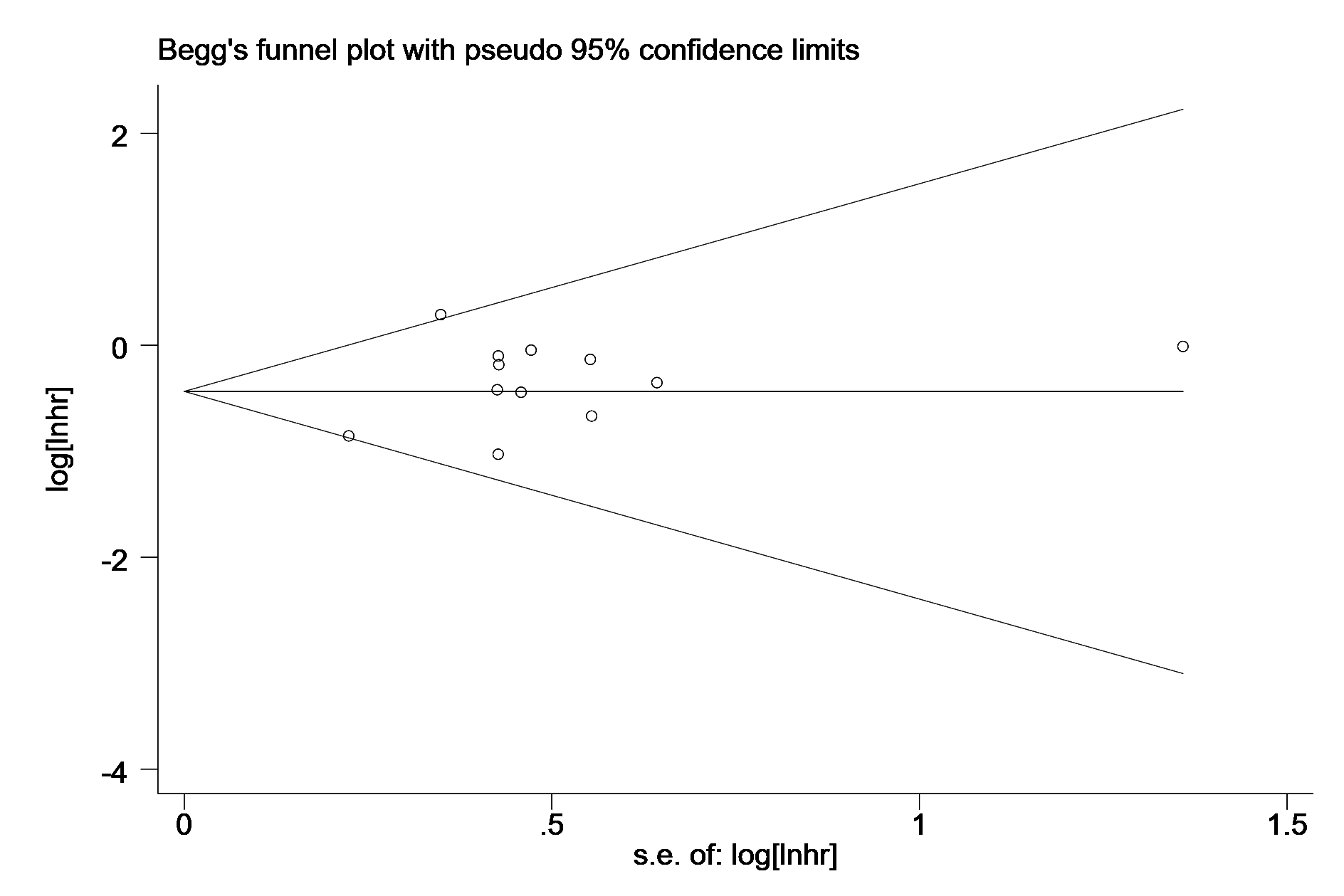


Fig S4. Funnel plots of publication bias for meta-analysis of sarcopenia for OS


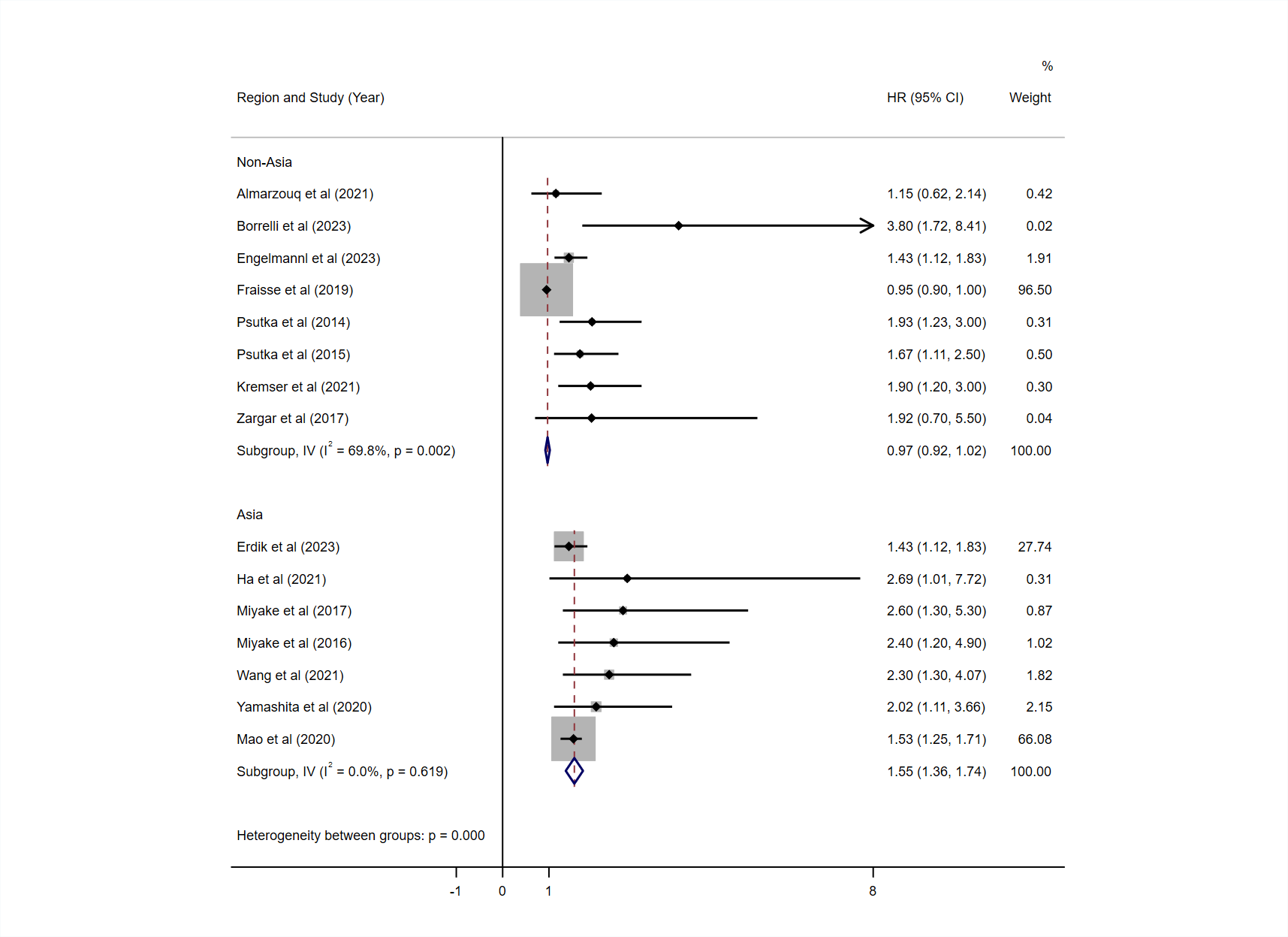


Fig S5. Regional impact of sarcopenia on OS


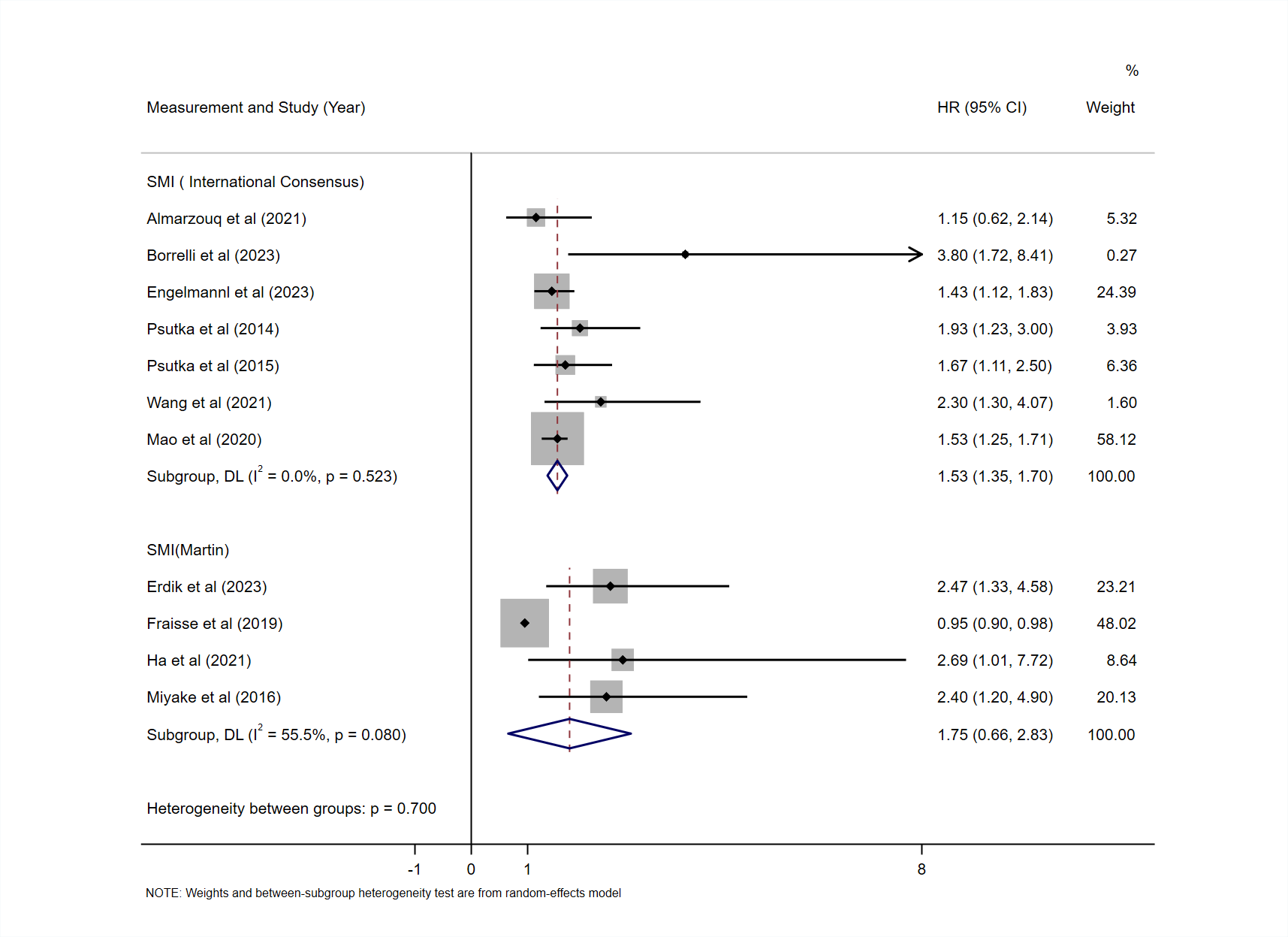


Fig S6. Measurement impact of sarcopenia on OS


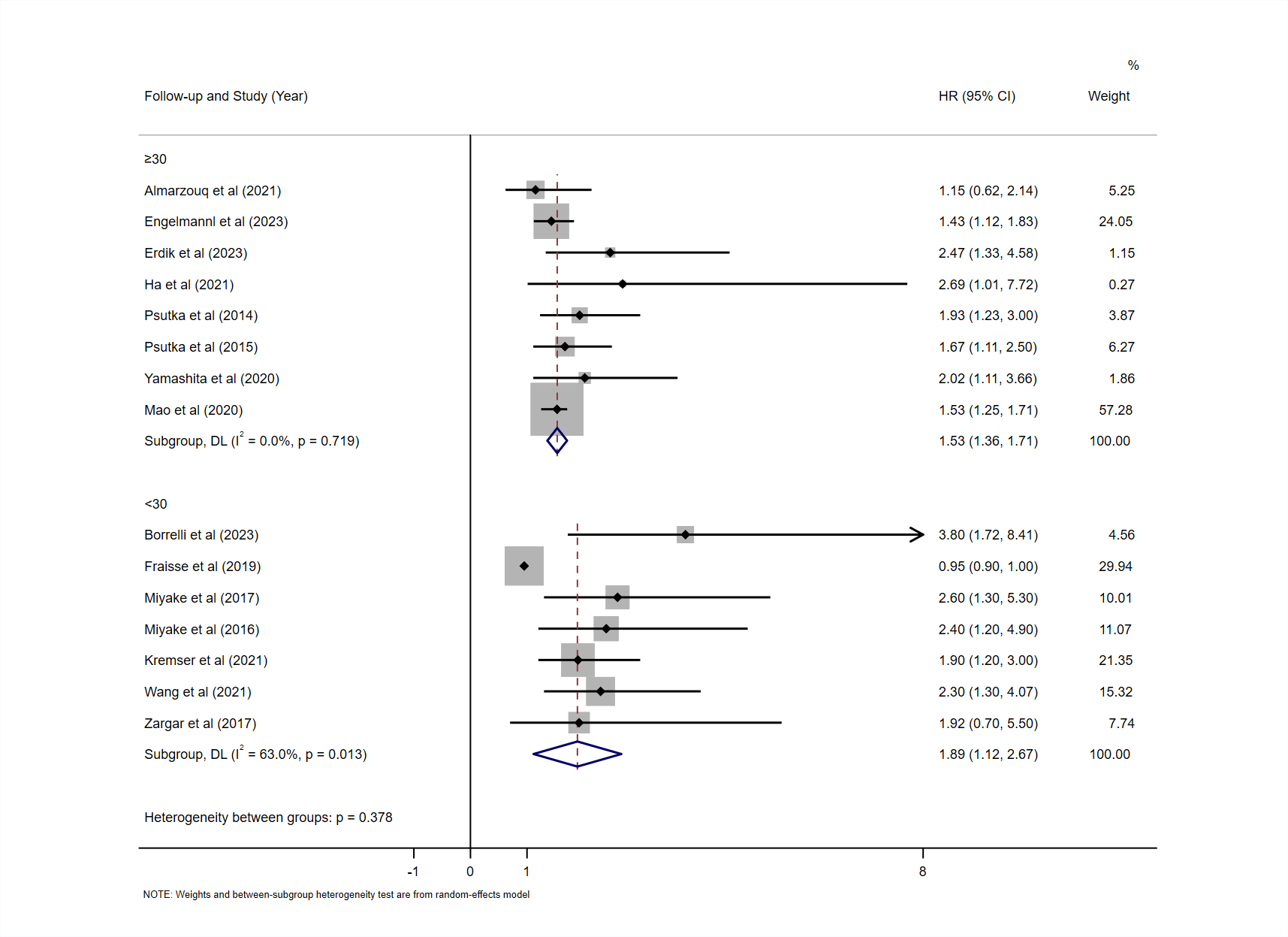


Fig S7. Follow-up impact of sarcopenia on OS


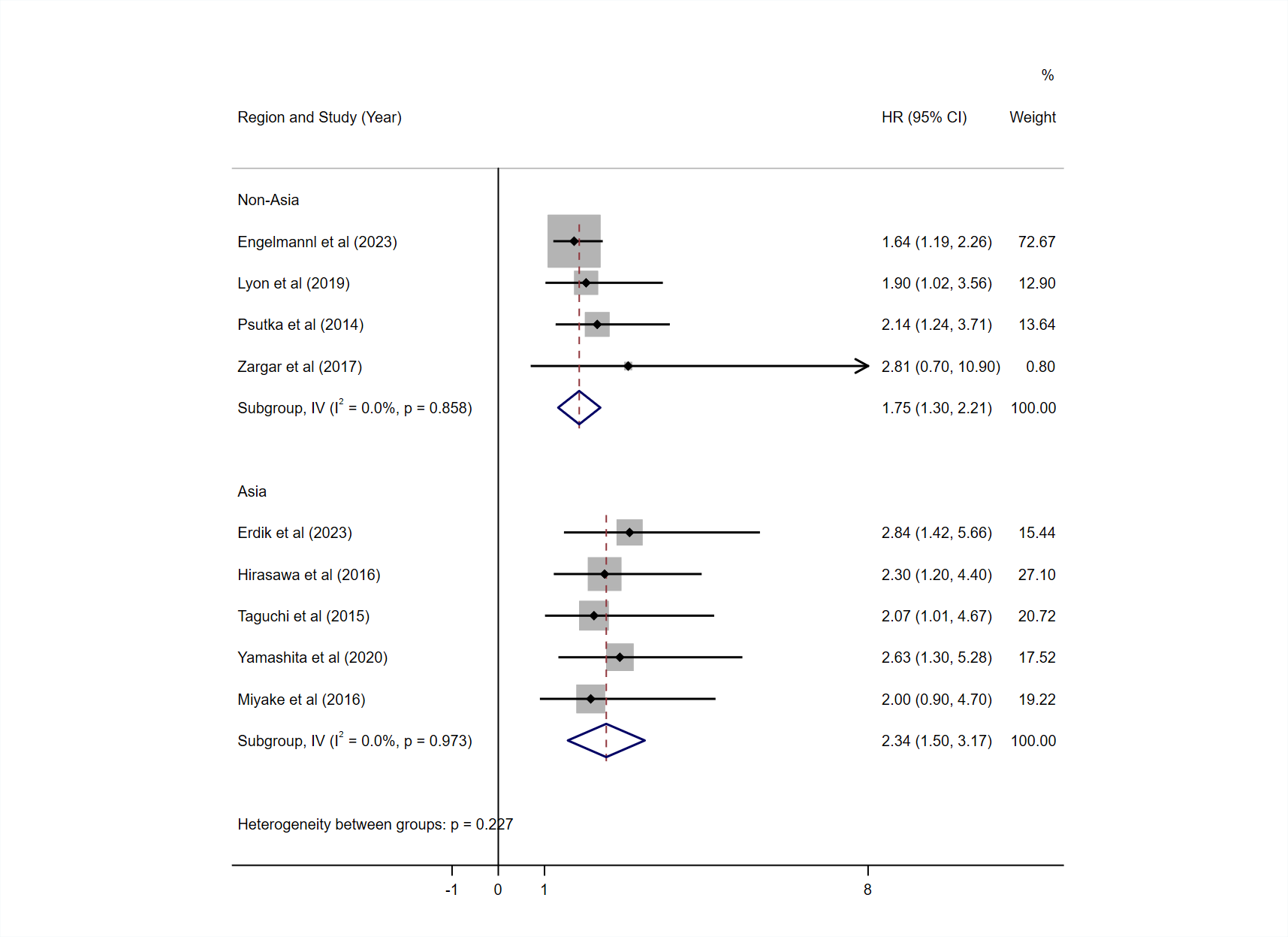


Fig S8. Regional impact of sarcopenia on CSS


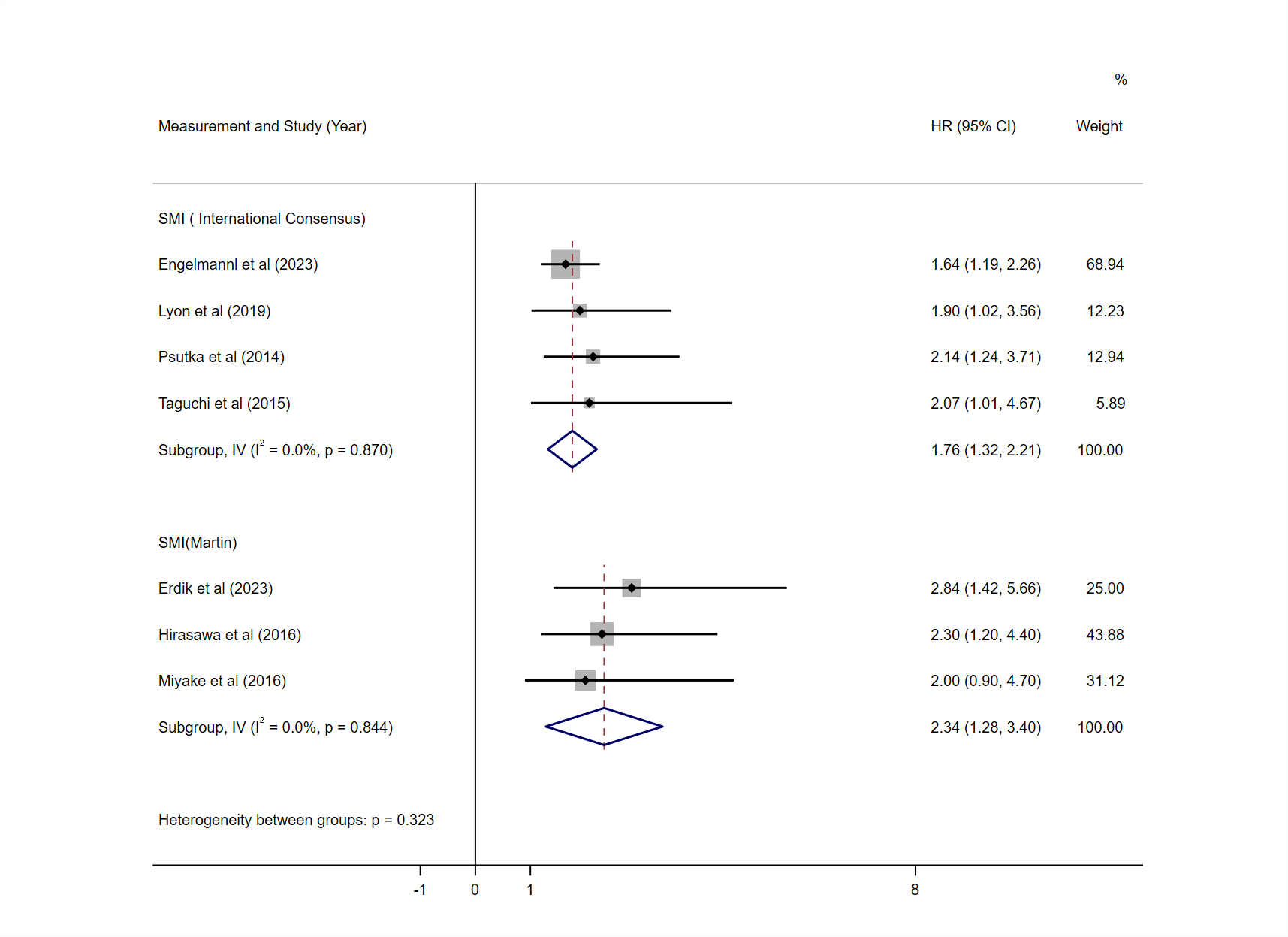


Fig S9. Measurement impact of sarcopenia on CSS


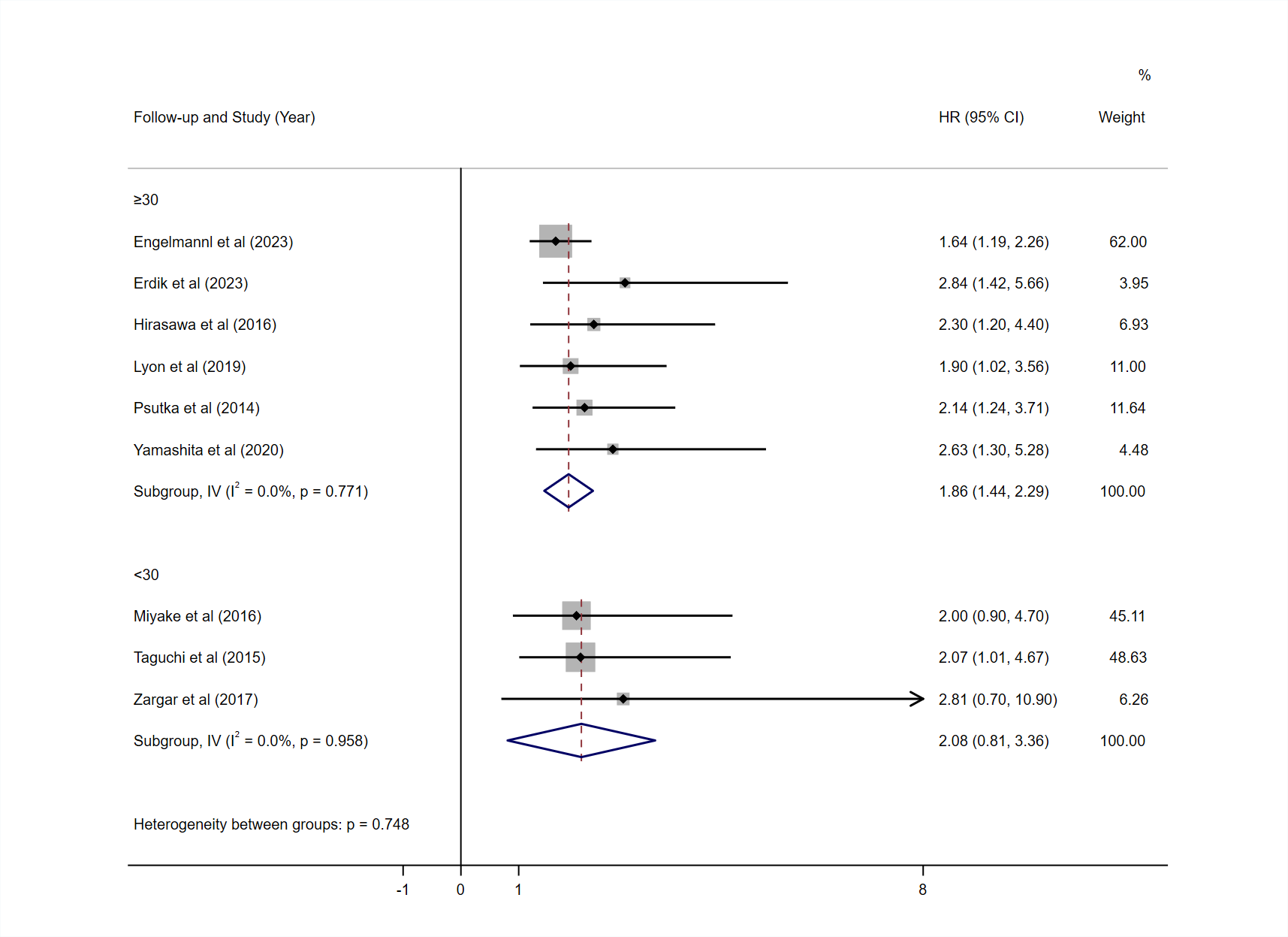


Fig S10. Follow-up impact of sarcopenia on CSS
